# A Multi-Institutional Retrospective Analysis of Toceranib Phosphate for Presumed or Confirmed Canine Aortic Body Chemodectomas

**DOI:** 10.3389/fvets.2021.635057

**Published:** 2021-02-05

**Authors:** Giovanna M. Coto, Margaret L. Musser, Melissa A. Tropf, Jessica L. Ward, Yeon-Jung Seo, Jonathan P. Mochel, Chad M. Johannes

**Affiliations:** ^1^Department of Veterinary Clinical Sciences, College of Veterinary Medicine, Iowa State University, Ames, IA, United States; ^2^Department of Statistics, University of Pittsburgh, Pittsburgh, PA, United States; ^3^SMART Pharmacology, Department of Biomedical Sciences, College of Veterinary Medicine, Iowa State University, Ames, IA, United States

**Keywords:** Palladia, heart base tumor, dog, chemotherapy, radiation, pericardiectomy

## Abstract

Aortic body tumors, specifically chemodectomas, are the second most common type of canine cardiac tumor; however, information about treatment is currently lacking. This study included dogs with a presumptive or definitive diagnosis of an aortic body chemodectoma that underwent treatment with toceranib phosphate. Cases were solicited via the American College of Veterinary Internal Medicine Cardiology, Internal Medicine, and Oncology listservs using an electronic survey. Cox multivariate analysis of factors potentially impacting survival time was completed. Twenty-seven (27) cases were included in analysis. The clinical benefit rate (complete remission, partial remission, or stable disease >10 weeks) was 89%. A median survival time of 478 days was found for those receiving toceranib alone (*n* = 14), which was not statistically different from those treated with additional modalities (521 days). No factors evaluated statistically impacted outcome. Further, prospective studies are warranted to evaluate the use of toceranib for the treatment of canine aortic body chemodectomas.

## Introduction

Aortic body tumors account for 8% of all histopathologically diagnosed canine cardiac tumors (1, 2). The most common type of aortic body tumor is the chemodectoma, which arises from the chemoreceptor cells of the ascending aorta (3). Chemodectomas usually present as a single mass at the base of the heart, most often occurring between the aorta and pulmonary artery, the aorta and right atrium, or between the pulmonary artery and left atrium (4, 5). They are non-functional and generally locally invasive tumors with a low metastatic propensity of up to 22% (2). The most common sites of metastatic disease include the regional lymph nodes, myocardium, lungs, liver, adrenal glands, and brain (3, 6).

Chemodectomas generally occur in older brachycephalic breeds (1, 2, 6–9). It is thought that chronic hypoxia, genetics, and other factors play a role in tumor pathogenesis (2, 6, 9). Chemodectomas are often an incidental finding on imaging or necropsy, but may present with a variety of clinical signs, most often secondary to either pericardial effusion with subsequent tamponade or physical compression of adjacent cardiac structures (2). Presumptive diagnosis of a chemodectoma is frequently made based on history, physical examination, thoracic radiographs, and echocardiogram findings. Fine needle aspirates for cytology or tissue biopsy for histopathology may be obtained for a definitive diagnosis when anatomically feasible (10).

There is limited data on the efficacy of current treatment options for aortic body chemodectomas. Surgical resection has been performed in select cases with variable results, but it has yet to be extensively studied (4, 11). Alternatively, pericardiectomy has been shown to significantly prolong survival times in dogs with heart base tumors, regardless of the presence or absence of pericardial effusion (7, 11). The utility of radiation therapy in the treatment of aortic body tumors has been evaluated in a small number of cases, with conformal or stereotactic radiation therapy resulting in clinical benefit, but with concern for potentially significant side effects (12, 13). The benefit of cytotoxic chemotherapy is thought to be limited, although it has yet to be fully elucidated (3).

Toceranib phosphate (Palladia®; Zoetis, Parsippany, New Jersey) has been used anecdotally to treat cases of presumed or confirmed aortic body tumors. Toceranib is a receptor tyrosine kinase inhibitor that was FDA approved for the treatment of canine mast cell tumors in 2009 (14). Inhibition of various tyrosine kinase receptors results in both an antiangiogenic effect as well as direct antitumor effect in a number of solid tumors in canines (15). While toceranib has been evaluated in a variety of tumor types, there is only one recently published retrospective study (16) and one case report to support its use in canine aortic body tumors (17).

The primary aim of this multi-institutional retrospective study was to evaluate the clinical benefit of toceranib in a population of dogs with presumed or confirmed aortic body chemodectomas, compared to the previously published literature, in an attempt to further support its use for this tumor type. A secondary aim was to evaluate the impact of additional treatment modalities (such as chemotherapy, radiation therapy, non-steroidal anti-inflammatory drugs, or surgery) on the clinical benefit rate of single-agent toceranib.

## Materials and Methods

### Data Collection

A multi-institutional retrospective analysis was performed. Cases of suspect aortic body chemodectomas treated with toceranib were solicited via the American College of Veterinary Internal Medicine (ACVIM) Cardiology, Internal Medicine, and Oncology listservs using an electronic survey (REDCap, Vanderbilt University, Nashville, TN, USA). Dogs with a presumptive diagnosis (based on a combination of clinical and imaging findings, as previously described (13)) or definitive diagnosis (histopathology and/or cytology) of an aortic body chemodectoma that were treated with toceranib and had adequate follow-up were eligible for inclusion. Data collected included signalment (age, sex, breed), presenting clinical signs, method of diagnosis, presence of metastatic disease, location of tumor, size of tumor in the longest diameter, cardiac structures noted to be compressed by the tumor, presence and type of arrhythmias, presence of cavitary effusion(s), toceranib dose (mg/kg), schedule and duration of treatment, best response, and adverse events. When available, reported adverse events were graded using the Veterinary Cooperative Oncology Group Common Terminology Criteria for Adverse Events v1.1 (18). Prior to treatment with toceranib, informed client consent was obtained for each patient per the requirements of the participating institution. No animal use oversight or other ethical approval was required for this retrospective study.

Due to the retrospective design, staging and follow-up intervals varied and were up to the discretion of the attending clinician. Best response to treatment was reported using the Response Evaluation Criteria for Solid Tumors v1.1 and was categorized as complete response, partial response, stable disease, or progressive disease (19). Clinical benefit to toceranib was defined as complete response, partial response or stable disease >10 weeks in duration (15). For those patients that achieved clinical benefit, the overall progression free interval was determined by calculating the time between the date of starting toceranib to the date that progressive disease was documented. Toceranib-specific survival was calculated using the Kaplan-Meier estimator from the first toceranib dose to death or last date of contact. Improvement or resolution of clinical signs or effusions were not part of the response to treatment assessment as clinical assessment is an inaccurate determination for response to therapy (19).

### Statistical Analysis

Ten covariates were considered to be potentially related to survival using univariate analyses including Kaplan–Meier methods and log-rank tests. The variables included in the model were: age, gender, breed, presence of cardiac compression, arrhythmias, pericardial effusion, pleural effusion, ascites, metastatic disease, and treatment. To assess the association between several risk factors and toceranib-specific survival time, a Cox proportional hazards regression model was built using a best subsets variable selection approach. Akaike and Bayesian/Schwartz information criteria (AIC, BIC or SBC) were then used to select an appropriate model. Statistical analyses were performed using commercial software, SAS 9.4 (SAS Institute, Cary, NC, USA) with results considered statistically significant at *p* < 0.05.

## Results

### Study Population

Twenty-eight (28) cases were collected via the ACVIM listservs from 12 institutions spanning 5 years (2013–2018). One was excluded due to lack of follow-up information. Dolichocephalic breeds were common, including mixed breeds, Pitbull mixes, and retrievers (*n* = 3 of each), Huskies and Yorkshire Terriers (*n* = 2 of each), and one each of Bichon Frise, Coonhound, Jack Russell, and Schnauzer breeds. Ten patients were considered to be brachycephalic including Boston Terriers (*n* = 6), Boxers (*n* = 2), and French Bulldogs (*n* = 2). One intact male, 13 castrated males, and 13 spayed females were evaluated. The median age at diagnosis was 10 years (range: 4–12 years).

Nine dogs (33%) presented with non-specific signs including lethargy, weight loss, and gastrointestinal signs. Four dogs (15%) were initially presented due to a new cough, three due to exercise intolerance (11%), two for syncope (7%), two for evaluation of a new heart murmur or arrhythmia (7%), one with cranial vena cava syndrome, one for a large cervical mass, one with increased respiratory rate and effort, and one with difficulty swallowing. Three dogs (11%) were not clinical at presentation.

Twenty-one dogs (78%) had a presumptive diagnosis of an aortic body chemodectoma based on breed predisposition and imaging (echocardiogram or CT). Six dogs (22%) had definitive antemortem diagnosis of chemodectoma via histology and/or cytology.

Eleven out of the 27 dogs (41%) had pericardial effusion present at diagnosis. Cardiac tamponade was present in 4/11 (36%) cases. Of the 11 dogs with pericardial effusion, five also had pleural effusion and ascites, and one had pleural effusion alone. Five additional dogs (18%) had pleural effusion without pericardial effusion while 11 patients (41%) had no effusions present. Five dogs (18%) had an arrhythmia documented via electrocardiography, including ventricular ectopy (*n* = 3), supraventricular ectopy (*n* = 1), and one unclassified arrhythmia.

Aortic body mass measurements and location were evaluated solely by echocardiogram in thirteen dogs (52%), thoracic computerized tomography (CT) in two dogs (7%), or chest radiographs in one dog. The remaining 11 dogs had a combination of diagnostic imaging modalities performed. Aortic body tumor size was recorded in 24 patients. The median longest diameter for the entire population using all measurement modalities was 5.4 cm (range: 1.8–12 cm). Two patients had tumor size measured with both echocardiogram and CT (5.5 and 4 cm, and 6.4 and 5.2 cm, respectively). The mass was most commonly located between the aorta and the pulmonary artery (10/27, 37%), but masses were also reported between the left atrium and the aorta (4/27, 15%), visualized in both locations (4/27, 15%), in other varied locations (8/27, 30%), and in one case tumor location was not reported.

Twenty-four dogs had an echocardiogram that allowed for evaluation of cardiac structure compression. Fourteen of these dogs (58%) were noted to have cardiac compression. The most common sites of compression included the pulmonary artery (*n* = 5), left atrium (*n* = 3) and right atrium (*n* = 1). Two of the 24 dogs had compression of other or unspecified cardiac structures, and three had a combination of cardiac structures compressed.

Twenty-six dogs were evaluated for metastatic disease at diagnosis or during treatment via a single imaging modality or a combination of thoracic radiographs (*n* = 22), abdominal ultrasound (*n* = 10), and CT (*n* = 12). Ten of these dogs (38%) had evidence of metastatic disease present, with the most common sites being to the lungs (*n* = 6) and regional lymph nodes (*n* = 4). Other metastatic sites included spleen, liver, kidney, and pancreas.

### Toceranib Phosphate Treatment

Toceranib was administered at a median dose of 2.55 mg/kg (range: 2.30–3.17 mg/kg) orally. Of the dogs for whom toceranib dose frequency was provided (26/27), 15 dogs received toceranib three times a week while the other 11 dogs received it every-other-day (every 48 h). The time between diagnosis and commencement of toceranib therapy varied (median 16, range: 0–608 days). All 27 patients were treated for non-resectable gross disease. Fourteen dogs (52%) received toceranib as a single agent (including 11 dogs with effusions), while the remaining 13 dogs received additional treatment as outlined in Table 1.

**Table 1:** Treatment and case outcomes for 27 dogs receiving toceranib phosphate for suspect aortic body chemodectomas.

**Treatments**	**Number of patients**	**Response**	**OST (days)**
Toceranib phosphate total	27		
Toceranib phosphate alone	14	CR (1)PR (3)SD (10)	467 93; LTF (245; 562) Median: 477 (*n* = 7); LTF (134; 578; 708)
Toceranib phosphate with:			
Pericardiectomy without pleural port	3	SD (2)PD (1)	LTF (852, 931) LTF (91)
NSAID	2	PRSD	279 153
Palliative radiation and NSAID	2	SD (2)	312; 521
Pericardiectomy with pleural port	1	SD	LTF (455)
Glucocorticoids	1	PD	LTF (320)
Chlorambucil and NSAID	1	PD	LTF (97)
Stereotactic radiation therapy	1	PR	LTF (594)
Pleural port alone	1	PR	LTF (178)
Stereotactic radiation and Pericardiectomy	1	SD	LTF (138)

*CR, complete remission; LTF, lost to follow-up; NSAID, non-steroidal anti-inflammatory drug; OST, overall survival time; PD, progressive disease; PR, partial remission; SD, stable disease. Individual OST are separated by semicolons*.

All dogs receiving toceranib were evaluated for response. Nine of these dogs presented with overt metastatic disease, one developed metastatic disease after starting treatment. Twenty-four (89%) achieved a clinical benefit, including complete response (*n* = 1), partial response (*n* = 6), and stable disease (*n* = 17). The remaining three dogs had progressive disease. Of the dogs that received toceranib and experienced a clinical benefit, 14 (one complete response, three partial response, 10 stable disease) received toceranib alone. The remaining 10 dogs received a combination of treatments as outlined in Table 1. Fifteen of the 24 dogs who achieved clinical benefit with toceranib treatment had complete medical records to allow for evaluation of progression free interval. The median progression free interval of those dogs was 240 days (range: 93–584).

**Figure 1:**
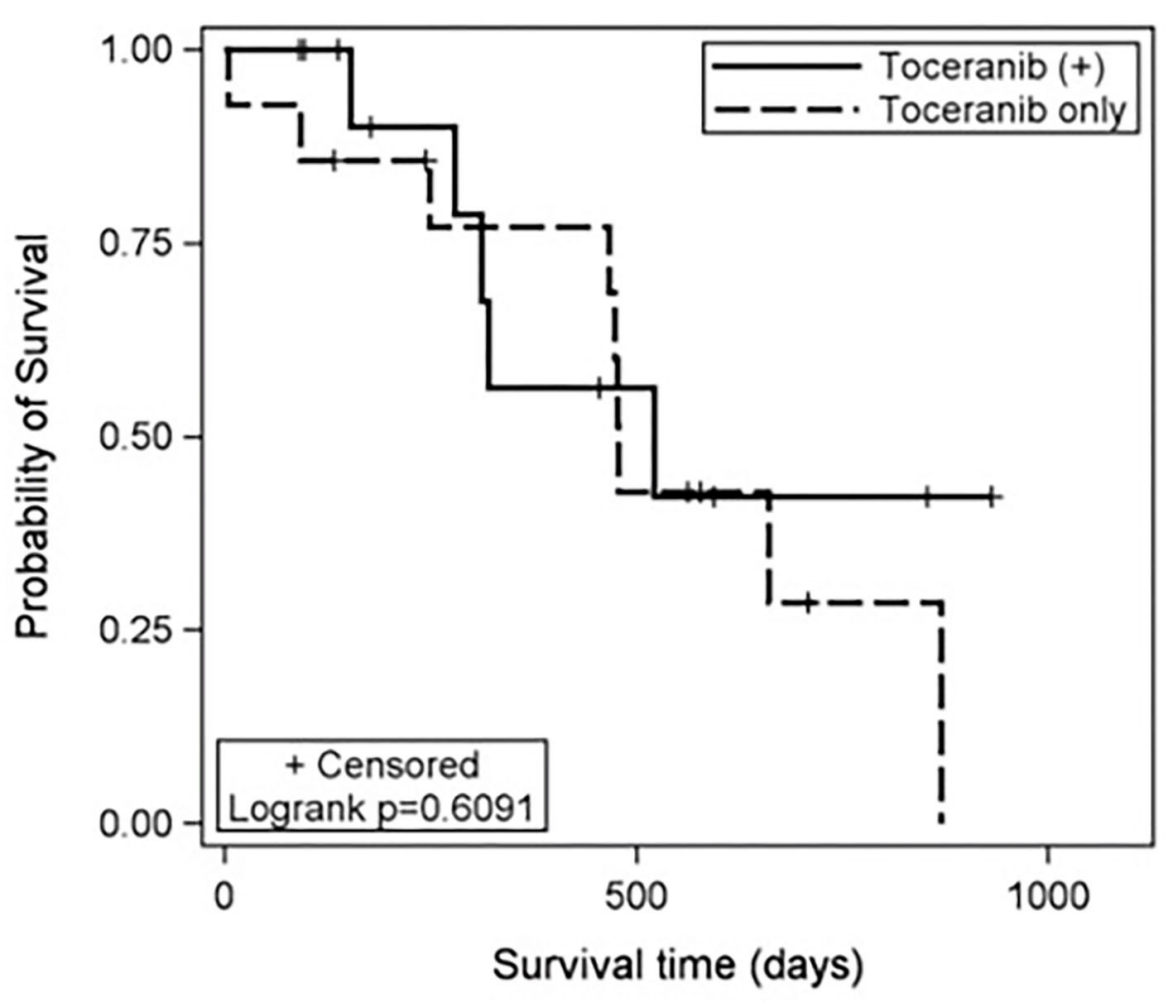
Kaplan-Meier estimates of the toceranib-specific survival time for patients treated with toceranib only (dashed line) and those treated with toceranib and additional modalities (solid line). The treatment effect was not statistically significant based on the log-rank test (*p* = 0.6091). In this study, the median toceranib-specific survival time for dogs treated with toceranib only was estimated to be 478 days and the median toceranib-specific survival of dogs treated with toceranib and additional modalities was estimated to be 521 days.

The median toceranib-specific survival time for those receiving toceranib alone (*n* = 14) was 478 days (range: 5–870 days). The median toceranib-specific overall survival time for those receiving toceranib and any additional treatment (*n* = 13) was 521 days (range: 91–931 days). This was not found to be statistically different (Figure 1). The one dog who received toceranib and achieved a complete response was dead at time of data collection with a toceranib-specific overall survival time of 468 days (Table 1). The survival time of the six dogs with a definitive diagnosis ranged from 402–662 days, with one dog being lost to follow-up at 455 days. Cox regression analysis revealed that none of the 10 confounding factors evaluated influenced risk of death.

Necropsy was performed in one dog and the presumptive chemodectoma diagnosis was confirmed.

### Toceranib Adverse Events

There were 33 adverse events reported in 17 of the 27 dogs (63%) receiving toceranib. The adverse events reported were consistent with previous reports from studies of toceranib use in canines, with diarrhea being the most common (*n* = 8 patients) (14, 20, 21). Other common adverse events consisted of decreased appetite (*n* = 6 patients), weight loss (*n* = 3 patients) and lethargy (*n* = 3 patients). There was a single case of grade two hypertension, which resolved after starting treatment with enalapril. Although the vast majority of toxicities were low grade, three grade three adverse events were reported (one nausea and two diarrhea). In addition, nine of the 17 dogs (53%) that experienced adverse events required dose adjustments (decrease in milligrams administered; change from every-other-day dosing to Monday, Wednesday, Friday; or both, at the discretion of the attending clinician). Adverse events triggering a dose adjustment included general gastrointestinal signs (lack of appetite, diarrhea), weight loss, grade I neutropenia, grade I azotemia, and grade II elevations in liver enzymes (alkaline phosphatase and alanine aminotransferase).

## Discussion

Canine aortic body chemodectomas are difficult to diagnose and known effective treatment options are limited. Toceranib is a small molecular weight tyrosine kinase inhibitor that competitively inhibits a number of tyrosine kinase receptors in the split kinase family (15, 22, 23). Inhibition results in both an antiangiogenic effect (via inhibition of receptors such as vascular endothelial growth factor receptor [VEGFR] and platelet derived growth factor receptor [PDGFR]), as well as direct antitumor effect (via inhibition of receptors such as c-Kit and rearranged during transfection kinase [RET]) in a number of solid tumors in canines (15, 22, 23).

Recently, a retrospective case series of 28 dogs with presumed aortic body chemodectomas treated with toceranib was published (16). An overall survival time of 823 days (range: 68–1190) was reported. This is numerically longer when compared to the overall survival time of 478 days found in the current study for dogs treated with toceranib alone. This difference may be related to several factors. In general, heart base masses are slow growing (16), thus, if the current population of patients was diagnosed later in the natural course of the disease, survival times will be erroneously shortened due to lead time bias. Unfortunately, it is not feasible to determine exactly when each tumor started to develop. The median aortic body tumor size in the current population was 5.4 cm, a substantial size that may indicate advanced disease. Median tumor size was not reported in the previous study, making comparisons impossible. The presence of metastatic disease is also indicative of more advanced disease. In the current population, nine dogs (32%) had metastatic disease present at diagnosis, with an additional dog developing metastatic disease during treatment. Historically, the metastatic rate is considered to be low (≤22%). The metastatic rate at presentation in the previous study was 29%. Data regarding the development of metastatic disease after starting toceranib was not available (16). Thus, based on tumor size and metastatic disease, it is plausible that the current population of dogs had more advanced disease when treatment commenced, resulting in a shorter overall survival time.

In addition, direct comparisons in median survival time between the two study populations are difficult, as median survival times appear to have been calculated differently. In the current study, median survival time was calculated from the start of toceranib to death or lost to follow-up. In the previous study, the methods for this calculation appear to be varied and may have commenced at diagnosis (16).

Similarly to the current study, the previous study found metastasis did not negatively impact response to toceranib (16). Furthermore, regardless of metastasis, both studies found a clinical benefit for 80–90% of patients treated with toceranib.

An additional case report describing toceranib use in a dog with a confirmed aortic body chemodectoma has been published (17). The patient received toceranib for 9 months, showing both clinical and radiographic improvement for the duration of that time. The patient experienced a progression free interval of 10 months, presenting with signs of recurrent right-sided congestive heart failure due to documented disease progression 1 month after discontinuation of treatment (17). The present study is consistent with these prior reports and suggests that toceranib may confer a clinical benefit, even with advanced disease.

It has been shown that carotid body paragangliomas in people, which are biologically similar to chemodectomas in dogs (5, 6) often overexpress VEGFR1, VEGFR2, PDGFRα, and PDGFRβ, making them potential targets for treatment with tyrosine kinase receptor inhibitors such as sunitinib (24). Clinical studies evaluating the use of sunitinib in patients with paragangliomas have reported a clinical response of at least 47% (25). Additionally, about 50% of human patients with malignant paragangliomas have been found to carry a hereditary germline mutation in the succinate dehydrogenase subunit B gene, which makes these patients particularly susceptible to treatment with sunitinib (26). While it is unknown if dogs with aortic body chemodectomas overexpress these receptors or harbor these mutations, one study did find a succinate dehydrogenase subunit D gene mutation in a dog with a paraganglioma (27), implying that toceranib, or similar small molecule inhibitors, may provide effective treatment for canine chemodectomas.

Study limitations include those inherent to a retrospective study: heterogenous population of clinical patients, small sample size, lack of follow-up protocol standardization, lack of a definitive diagnosis in most cases, lack of pathologically confirmed metastatic disease, and assumption of cause of death due to lack of complete necropsy in most cases.

Presumptive diagnosis of chemodectoma in this study was based on a combination of signalment (brachycephalic breed) and/or echocardiographic location of the mass (located in the area of the heart base), as previously described (13). The sensitivity of echocardiography for detecting cardiac mass lesions is generally good (up to 82%) (28–30). A recent retrospective study showed that in patients with heart base tumors, presumptive echocardiographic diagnosis of chemodectoma, ectopic thyroid carcinoma, or lymphoma was correct 78% of the time, with the other 22% of cases being ultimately diagnosed as undifferentiated carcinomas (31). Thus, it is possible that some patients in the current population, may have been erroneously diagnosed, impacting the study conclusions and emphasizing the importance of obtaining a definitive diagnosis via cytology or histopathology prior to commencement of treatment.

While survival comparison between dogs with a definitive chemodectoma diagnosis compared to those presumptively diagnosed would have been ideal, the small number of cases prohibited statistical evaluation. This is potentially pertinent as toceranib has been evaluated for the treatment of other heart base tumors, including thyroid carcinoma and hemangiosarcoma. Toceranib has been shown to confer a clinical benefit for canine thyroid carcinoma, with a similar survival time to the current study (563 days) (15, 32). However, toceranib conferred no clinical benefit for canine splenic hemangiosarcoma (22, 33); it is presumed that cardiac hemangiosarcoma would be similar. The overall survival time of dogs with cardiac hemangiosarcoma is significantly shorter (7.1 days without treatment, 42 days with surgery alone) compared to the survival time reported in the current study (3). Thus, the impact on the findings described herein of a misdiagnosis that happens to respond to toceranib is likely to be small but must be acknowledged.

Despite these limitations, this study supports the use of toceranib as a treatment consideration for presumptively diagnosed chemodectomas. Toceranib was well-tolerated and provided clinical benefit to 89% of patients. As there are now two retrospective evaluations of toceranib in dogs with presumed chemodectomas reporting similar findings, the argument for further evaluation in controlled prospective studies is strengthened and necessary to fully assess the clinical utility of toceranib in dogs with confirmed chemodectomas.

## Data Availability Statement

The raw data supporting the conclusions of this article will be made available by the authors, without undue reservation.

## Ethics Statement

Ethical review and approval was not required for the animal study as it was a retrospective study. Prior to treatment with toceranib, informed client consent was obtained for each patient per the requirements of the participating institution.

## Author Contributions

GC and MM prepared the digital survey with input from MT, JW, and CJ for accurate data collection. Data was reviewed and descriptive statistics were prepared by GC, MM, MT, JW, Y-JS, JM, and CJ. Full statistical analysis was completed by Y-JS and JM. Manuscript preparation was completed by GC and MM and reviewed by all authors.

## Conflict of Interest

CJ is a former employee of Pfizer Animal Health. The remaining authors declare that the research was conducted in the absence of any commercial or financial relationships that could be construed as a potential conflict of interest.
